# Apolipoprotein E4 contributes to Alzheimer’s disease neuropathology in Down syndrome

**DOI:** 10.1002/alz.091727

**Published:** 2025-01-03

**Authors:** Breanna Dooling, Rose Summers, Daphne Quang, Molishree Joshi, Hector Esquer, Huntington Potter, Noah R Johnson

**Affiliations:** ^1^ University of Colorado Anschutz Medical Campus, Aurora, CO USA; ^2^ University of Colorado Alzheimer’s and Cognition Center, Aurora, CO USA; ^3^ Linda Crnic Institute for Down Syndrome, Aurora, CO USA; ^4^ Skaggs School of Pharmacy and and Pharmaceutical Sciences, Aurora, CO USA

## Abstract

**Background:**

Adults with Down syndrome (DS) develop Alzheimer’s disease (AD) brain pathology by their 40s due to triplication of the amyloid precursor protein (APP) gene on chromosome 21, and most develop clinical symptoms by age 50‐60. Inheritance of the apolipoprotein E (apoE) ε4 allele (*APOE4*) is the strongest risk factor for AD besides age, whereas the ε3 allele (*APOE3*) does not change AD risk. The *APOE4* genotype is associated with earlier and more rapid cognitive decline in both typical AD and DS‐associated AD (DS‐AD); however, understanding of the associated mechanisms is lacking.

**Method:**

We developed cerebral organoids (COs) from trisomy 21 (T21) and disomy 21 (D21) *APOE3/3* human induced pluripotent stem cell (hiPSC) lines. We then used CRISPR‐Cas9 editing to modify our hiPSCs from an *APOE3/3* genotype to *APOE4/4* in DS hiPSCs and in matched control hiPSCs disomic for chromosome 21. We differentiated these hiPSCs into COs as well as into monolayer cultures of astrocytes and microglia.

**Result:**

There was a significant decrease in size from D21 COs to T21 COs. T21 COs also accumulated more apoE and Aβ than D21 COs and at earlier timepoints. T21 and *APOE4/4* each resulted in increased microglial size and decreased microglial roundness. T21 astrocytes accumulated more apoE than D21 astrocytes. T21 *APOE4/4* astrocytes exhibited increased cell death compared to T21 *APOE3/3* astrocytes at an early timepoint.

**Conclusion:**

*APOE4/4* further accelerates and enhances AD neuropathologies in people with DS. Understanding the molecular and cell type‐specific changes caused by *APOE4* as compared to *APOE3* in DS‐AD will aid in the development of more targeted and proactive therapies to alleviate dementia risk in individuals with DS.

